# Comparison of quality of life in patients with peripheral arterial disease caused by atherosclerosis obliterans or Buerger’s disease

**DOI:** 10.5830/CVJA-2014-017

**Published:** 2014-06

**Authors:** Rojbin Karakoyun, Cüneyt Köksoy, Zeynep Şener, Umut Gündüz, Barış Karakaş, Mustafa Karakoyun

**Affiliations:** Division of Vascular Surgery, Department of General Surgery, Faculty of Medicine, Ankara University, Ankara, Turkey; Division of Vascular Surgery, Department of General Surgery, Faculty of Medicine, Ankara University, Ankara, Turkey; Division of Vascular Surgery, Department of General Surgery, Faculty of Medicine, Ankara University, Ankara, Turkey; Department of Surgery, Antalya Training and Research Hospital, Antalya, Turkey; Department of Surgery, Antalya Training and Research Hospital, Antalya, Turkey; Department of Radiology, Veni-Vidi Hospital, Diyarbakir, Turkey

**Keywords:** Buerger’s disease, atherosclerosis obliterans, quality of life

## Abstract

**Objective:**

Buerger’s disease and atherosclerosis obliterans (ASO) are two peripheral arterial diseases (PAD) that are frequently encountered. The aim of this study was to compare quality of life (QOL) in patients with Buerger’s disease and ASO.

**Methods:**

We prospectively followed 86 patients who were admitted to our hospital due to ASO or Buerger’s disease. Their ischaemia was evaluated according to the clinical category chronic limb ischaemia at the time of hospital admission and at six and 12 months. The QOL was measured at the time of hospital admission and at six and 12 months with the Short Form Health Status Survey (SF-36) and Vascular Quality of Life Questionnaire (VASCUQOL).

**Results:**

A total of 86 patients with ASO or Buerger’s disease (47 and 39, respectively) were included in the study. Pain parameters from both SF-36 and VASCUQOL scores were lower in patients with Buerger’s disease at the time of hospital admission and at six months. The impairment in QOL was found to be proportional to the extent of chronic limb ischaemia. Conversely, when patients with critical limb ischaemia were evaluated, no difference was observed between those with ASO or Buerger’s disease in terms of QOL. Amputations were found to have a negative effect on quality of life.

**Conclusion:**

Buerger’s disease had a more pronounced negative effect on QOL than ASO, particularly in terms of pain score. When critical limb ischaemia was considered, ASO and Buerger’s disease impaired quality of life at the same rate.

## Abstract

Vascular diseases are well studied today because of the relationship between arterial age and human age, the close connection between aging and arterial diseases, and the fact that the majority of deaths result from diseases of the vascular system. Among vascular diseases, chronic occlusive arterial disease of the lower extremities is of great importance in terms of mortality and morbidity. An increase in the incidence of peripheral arterial diseases is also a consequence of the gradually increasing size of the elderly population.^1^

The treatment strategy for patients should not be intended only for the prevention of extremity loss in peripheral arterial diseases (PAD), but also for the determination and treatment of risk factors, thus resulting in increased quality of life. Nowadays, the objective determination of quality of life, and evaluation of the association between quality of life and treatment methods in PAD is of great importance.^1^-^3^

Buerger’s disease is a PAD that is frequently encountered and causes significant loss of the extremities. Generally, although its pathological characteristics are well described, there is inadequate information about its impact on daily life and general health levels of patients, and about the difference in terms of quality of life between patients with Buerger’s disease and atherosclerosis obliterans (ASO), which are the most significant causes of PAD.

These two types of PAD show different aetiology, age of appearance and co-morbidity. These differences may influence the patient’s quality of life differently. Based on our clinical observations, we assumed the quality of life in patients with Buerger’s disease may be worse than that of patients with ASO.

Although studies evaluating quality of life in PAD have been carried out, there is, to our knowledge, no study that evaluates quality of life in patients with Buerger’s disease, or a comparison of quality of life in patients with ASO and Buerger’s disease, which are the most significant chronic occlusive arterial diseases. The objective of our study was to compare the quality of life in patients with ASO and Buerger’s disease.

## Methods

This prospective study included patients with ASO and Buerger’s disease who were admitted to the Department of Vascular Surgery, University of Ankara between 2008 and 2010. Those patients with chronic lower limb ischaemia due to ASO or Buerger’s disease were evaluated in terms of symptoms, risk factors and accompanying vascular or non-vascular diseases. An arterial examination of the lower extremity, and an ankle/brachial pressure index (ABI) and arterial duplex examination of the patients were then performed.

Patients with ASO who matched the criteria for inclusion in this study were those with atherosclerotic lower limb ischaemia at the time of admission, and patients with lower extremity involvement. Patients with Buerger’s disease who matched the criteria for inclusion in this study were those with lower extremity involvement, and patients with Buerger’s disease, diagnosed according to Shionoya’s criteria,^4^ at the time of admission. These criteria included smoking history; onset before the age of 50 years; infra-popliteal arterial occlusions; either upper limb involvement or phlebitis migrans; and absence of atherosclerotic risk factors other than smoking. Written informed consent was obtained from all study participants.

At the time of hospital admission, the patients’ ischaemia was evaluated according to the clinical categories of chronic limb ischaemia (Rutherford classification^5^) by inviting patients back to the hospital at six and 12 months following the first admission. Patients’ quality of life was evaluated from the Short Form Health Status Survey (SF-36), which provides a measurement of change in physical components, including physical function, physical status, bodily pain and general health, as well as mental components, including mental health, emotional status, social function and vitality.

The impact of PAD was also evaluated from the Vascular Quality-of-Life Questionnaire (VASCUQOL), which consists of pain, symptoms, activities, social well-being and emotional wellbeing domains.^6^ The questionnaries were applied by a research staff member who had no information on these patients.

Following the measurement of quality of life, treatment modalities including surgery or medical therapy, where necessary and appropriate, were initiated. In general, endovascular and bypass procedures were preferred for ASO patients with critical ischaemia and unbearable claudication. Risk-factor reduction and medical treatments were used for all ASO patients. For Buerger’s disease patients with critical ischaemia, the target vessel was evaluated for distal bypass and smoking cessation was advised. Distal bypass was used for patients where appropriate.

Sympathectomy, infusion of prostaglandin (PGE2), medical treatment and wound care were used for patients in whom distal bypass was inappropriate or in those who had failed bypass. In the presence of necrotic tissue, minor or major amputations were performed. All parameters evaluated at baseline were also repeated at six and 12 months post admission.

## Statistical-analysis

Comparisons between groups were made using Pearson’s chi-square test for categorical variables and independent *t*-tests for continuous variables. A *p*-value less than 0.05 was accepted as statistically significant. Statistical analyses were carried out using SPSS for Windows 15.0 (SPSS Inc., Chicago, Illinois, USA).

## Results

A total of 86 patients, 47 with ASO and 39 with Buerger’s disease, were included in the study. Demographic characteristics and additional diseases at the time of admission of the patients are shown in Table 1. The rate of smoking was statistically significantly higher in Buerger’s disease patients than in those with ASO (*p* = 0.002). The frequency of diabetes and hypertension was significantly higher in ASO patients than in those with Buerger’s disease (*p* < 0.001, *p* < 0.001 respectively).

**Table 1:** Demographic characteristics, additional diseases and amputation rates of patients

	*Atherosclerosis (n = 47)*	*Buerger’s disease (n = 39)*	p*-value*
Age (years)	60.28	47.77	ns
Gender			
Male (%)	80.9	94.9	ns
Female (%)	19.1	5.1	ns
Smoker (%)	59.6	94.9	0.002*
Non-smoker (%)	40.4	5.1	ns
Additional diseases			
Diabetes (%)	49.1	9.6	< 0.001*
Hypertension (%)	49.9	7.6	< 0.001*
Obesity (%)	4.4	5.4	ns
ABI (mean)	0.42	0.40	ns
Affected extremity (right–left) (%)	51–41	25–41	ns

ABI = ankle/brachial pressure index. *Statistically significant value.

## Vascular involvement at the time of admission

In the ASO group, claudication was identified in 23 (48.9%) patients, pain at rest occurred in six (12.8%), and ischaemic wound symptoms were observed in 18 (38.3%) patients. In the Buerger’s disease group, claudication was identified in one (2.6%) patient, pain at rest occurred in six (15.4%), and ischaemic wound symptoms were observed in 32 (82%) patients at the time of admission to hospital. The rate of ischaemic wound symptoms in patients with Buerger’s disease was statistically significantly higher than in the ASO group (*p* = 0.001).

There were statistically significant differences between the groups in anatomical localisation of ischaemic wounds, which were determined with invasive and non-invasive methods. In Buerger’s disease patients, the level of disease was observed to be mostly at the popliteo-crural level. In the ASO group, aortoiliac involvement was present in 18 (38.2%) patients, femoropopliteal involvement was found in 18 (38.2%), and popliteocrural involvement was present in 11 (23.4%) patients. In the Buerger’s disease group, aorto-iliac involvement was present in two (5.1%) patients, femoro-popliteal involvement was found in 10 (25.6%), and popliteo-crural involvement was present in 27 (69.2%) patients (*p* < 0.001).

According to chronic limb ischaemia criteria, Buerger’s disease patients were found to be in an advanced stage at the beginning of the study (Table 2). A total of 38.3% of patients in the ASO group and 76.9% of those in the Buerger’s disease group were in category 5 (*p* < 0.001).

**Table 2:** Comparison of the two groups in terms of chronic limb ischaemia criteria

*Category*	*Atherosclerosis, n (%)*	*Buerger’s disease, n (%)*	*Total, n (%)*	p*-value*
Category 1	2 (4.3)	0 (0)	2 (2.3)	ns
Category 2	9 (19.1)	0 (0)	9 (10.5)	ns
Category 3	12 (25.5)	1 (2.6)	13 (15.1)	ns
Category 4	6 (12.8)	6 (15.4)	12 (14)	ns
Category 5	18 (38.3)	30 (76.9)	48 (55.8)	< 0.001*
Category 6	0 (0)	2 (5.1)	2 (2.3)	ns

*Statistically significant value.

The two groups were compared in terms of number of operations performed at the time of hospital admission, and a statistically significantly higher number of vascular operations was observed in the ASO patients compared with the Buerger’s disease patients. Peripheral vascular surgery was performed in a total of 30 (63.8%) patients in the ASO group and nine (23.1%) in the Buerger’s disease group (*p* < 0.001). When early vascular complications related to vascular interventions were compared, no significant differences were observed between the two groups in terms of patency and thrombosis rates (data not presented).

The rate of minor and major amputations undertaken in the contralateral leg was 4.2 and 4.2%, respectively in ASO patients. The corresponding rates in Buerger’s disease patients were 20.5 and 10.3%, respectively. The rate of both minor and major amputations in the contralateral leg was significantly higher in Buerger’s disease patients (*p* = 0.027, *p* = 0.027, respectively).

The rate of minor amputations undertaken in the leg with a lesion was 4.2% in ASO patients and 35.9% in Buerger’s disease patients; this difference was statistically significant (*p* < 0.001). The rate of amputation within one year of hospital admission was found to be 23.4% in ASO patients and 48.7% in Buerger’s disease patients (*p* < 0.05). There was no mortality in either group during the one-year follow-up period.

## Results of SF-36

The two groups were evaluated using the SF-36 score measuring quality of life. A statistically significant difference was found between the two groups in terms of pain at the time of hospital admission and at six months post admission (*p* = 0.02, *p* = 0.001 respectively). Pain in Buerger’s disease patients was observed to be more pronounced; however, this difference disappeared at 12 months (Table 3).

**Table 3:** Evaluation of SF-36 between the groups

	*First evaluation*			*6-month follow up*			*12-month follow up*		
*Quality-of-life variables*	*Atherosclerosis (n = 47)*	*Buerger’s disease (n = 39)*	p*-value*	*Atherosclerosis (n = 33)*	*Buerger’s disease (n = 33)*	p*-value*	*Atherosclerosis (n = 22)*	*Buerger’s disease (n = 25)*	p*-value*
Physical function	27.3 ± 3.6	26.9 ± 4.2	0.94	53.4 ± 6.0	47.9 ± 5.5	0.50	67.9 ± 7.3	56.9 ± 6.2	0.26
Physical status	9.5 ± 3.9	3.8 ± 2.6	0.25	60.6 ± 8.3	44.2 ± 7.7	0.15	70.4 ± 9.6	50.5 ± 9.3	0.14
Social function	34.7 ± 3.7	26.4 ± 4.0	0.12	58.7 ± 5.2	47.7 ± 5.6	0.15	75.5 ± 7.5	70.7 ± 6.8	0.63
Body pain	30.1 ± 3.4	19.9 ± 2.7	0.02*	68.5 ± 5.0	41.3 ± 5.8	0.001*	78.6 ± 6.9	64.3 ± 8.1	0.19
Mental health	52.9 ± 3.6	50.6 ± 3.6	0.65	60.7 ± 4.2	54.2 ± 4.4	0.28	72.1 ± 3.7	65.2 ± 3.9	0.21
Emotional status	18.6 ± 4.9	11.9 ± 4.6	0.33	60.5 ± 7.9	49.3 ± 7.7	0.31	74.2 ± 9.2	56.1 ± 9.2	0.17
Vitality	36.0 ± 3.2	36.2 ± 3.3	0.97	52.6 ± 4.1	47.5 ± 4.2	0.39	62.7 ± 5.2	56.0 ± 4.0	0.31
General health	43.2 ± 2.5	37.4 ± 2.4	0.10	55.7 ± 3.2	49.3 ± 3.0	0.15	64.1 ± 4.7	62.1 ± 4.1	0.74

Values are median ± SE. *p*-values are between-group comparisons. *Statistically significant value.

The SF-36 scores for patients with peripheral vascular disease (irrespective of group) were compared according to category of chronic limb ischaemia. The quality of life of patients whose level of chronic limb ischaemia was category 5 and over at the time of the first admission was worse in terms of pain, general health, social function, emotional status and mental health than that of patients with SF-36 scores less than category 5. These parameters were found to be statistically significant (*p* = 0.02, *p* = 0.02, *p* = 0.02, *p* = 0.03, *p* = 0.04, respectively) (Table 4). The difference in quality of life according to category of the SF-36 questionnaire disappeared at six and 12 months.

**Table 4:** Evaluation of SF-36 according to category level for independent groups

	*First evaluation*			*6-month follow up*			*12-month follow up*		
*Quality-of-life variables*	*Category ≤ 4 (n = 36)*	*Category ≥ 5 (n =50)*	p*-value*	*Category ≤ 4 (n = 24)*	*Category ≥ 5 (n = 43)*	p*-value*	*Category ≤ 4 (n = 22)*	*Category ≥ 5 (n = 25)*	p*-value*
Physical function	28.3 ± 4.0	26.3 ± 3.7	0.71	58.7 ± 6.8	46.1 ± 4.9	0.61	63.7 ± 8.6	61.1 ± 5.7	0.81
Physical status	7.6 ± 4.3	6.5 ± 3.0	0.82	59.8 ± 9.4	48.1 ± 7.2	0.72	58.3 ± 8.6	60.7 ± 8.5	0.79
Pain	33.8 ± 3.6	19.6 ± 2.5	0.02*	63.9 ± 6.3	49.7 ± 5.4	0.55	73.0 ± 7.7	69.7 ± 7.4	0.78
General health	46.8 ± 2.7	36.0 ± 2.1	0.02*	57.0 ± 3.5	49.9 ± 2.8	0.81	63.0 ± 5.1	63.0 ± 3.9	0.92
Vitality	38.4 ± 3.5	34.4 ± 3.0	0.39	56.5 ± 4.0	46.4 ± 3.9	0.77	61.6 ± 5.5	57.6 ± 4.0	0.83
Social function	38.0 ± 4.4	25.7 ± 3.3	0.02*	62.8 ± 5.9	47.7 ± 4.9	0.54	73.1 ± 8.3	72.8 ± 6.4	0.81
Emotional status	24.3 ± 6.3	9.3 ± 3.5	0.03*	65.4 ± 8.3	48.9 ± 7.2	0.56	67.3 ± 9.5	62.9 ± 8.6	0.75
Mental health	58.0 ± 3.9	47.5 ± 3.3	0.04*	65.2 ± 4.6	53.0 ± 3.8	0.78	68.9 ± 4.7	68.1 ± 3.3	0.91

Values are median ± SE. *p*-values are between-group comparisons. *Statistically significant value.

Using the SF-36, when ASO and Buerger’s disease patients were divided into subgroups in terms of category of chronic limb ischaemia, no significant difference was determined for this parameter. However, when patients with advanced PAD in category 5 and over were compared with patients with chronic ischaemia of category 5 and over, there was no statistically significant difference between these groups (data not presented). From these data, quality of life in patients with both ASO and Buerger’s disease was negatively affected after critical ischaemia developed in the leg.

## Results of VASCUQOL

Quality of life was evaluated with VASCUQOL at the time of hospital admission, and at six and 12 months’ follow up. Statistically significant differences were found between both groups in terms of pain at the time of hospital admission. Statistically significant differences were also found between both groups in terms of pain, symptom level and social status at six months (*p* = 0.008, *p* = 0.04, *p* = 0.02, *p* = 0.02, respectively). Pain was observed to be more explicit and social status more impaired in Buerger’s disease patients; however, this difference disappeared at 12 months (Table 5).

VASCUQOL scores of patients with PAD (irrespective of group) were compared according to the clinical categories of chronic limb ischaemia (Rutherford classification4). Patients whose category level was 5 and over were observed to be significantly worse in terms of total VASCUQOL score, symptom level and social status at the time of the first admission, as well as symptom level and social status at six months (Table 6).

In terms of total VASCUQOL score, when patients with advanced PAD in only category 5 and over were compared with patients with chronic ischaemia of category 5 and over, there were no statistically significant differences between the groups in at the time of hospital admission or at six and 12 months (*p* = 0.602, *p* = 0.347, *p* = 0.839, respectively) (data not presented).

## Impact of amputation on quality of life

The impact of limb amputations within one year of hospital admission on quality of life was evaluated. According to the SF-36 score, at admission, amputation negatively affected pain in patients with both ASO and Buerger’s disease (*p* = 0.003). The amputation also negatively affected physical function, physical status, pain, general health, social functions and emotional status in these patients at six months (*p* = 0.002, *p* = 0.007, *p* = 0.001, *p* = 0.009, *p* = 0.005, *p* = 0.001, respectively) and at 12 months (*p* = 0.002, *p* = 0.007, *p* = 0.001, *p* = 0.009, *p* = 0.005, *p* = 0.001, respectively). Additionally, amputation was found to negatively affect mental status in the ASO patients at 12 months (*p* = 0.011). The total VASCUQOL scores were significantly lower in amputees with ASO and Buerger’s disease at six and 12 months (*p* = 0.039, *p* = 0.001, respectively) than the SF-36 scores.

Difference in quality of life in ASO and Buerger’s disease patients who were not amputees was evaluated. The total VASCUQOL score was found to be lower in Buerger’s disease patients at the time of hospital admission and at six months (*p* = 0.032, *p* = 0.005, respectively) than in ASO patients.

Difference in quality of life between amputees with Buerger’s disease and ASO was also evaluated. No significant differences in quality of life were observed at admission or at six and 12 months in these groups (*p* = 0.84, *p* = 0.48, *p* = 0.32, respectively). These results could have been related to the fact that the source of pain had been amputated in these ASO and Buerger’s disease patients, therefore no difference in quality of life would be expected. However, quality of life was observed to be significantly worse in non-amputee Buerger’s disease patients than ASO patients.

## Discussion

Peripheral arterial diseases are high-morbidity diseases that have a negative effect on quality of life.^7^-^9^ Although there are numerous studies on quality of life in patients with PAD, or comparing quality of life between patients with PAD and other cardiovascular diseases,10 to our knowledge, there is no study that compares quality of life between the different categories of PAD, namely ASO and Buerger’s disease. This study is therefore the first to undertake such a comparison.

According to our study, in general, although Buerger’s disease had a more pronounced negative effect on quality of life, particularly in terms of pain score, both ASO and Buerger’s disease impaired quality of life to a similar degree when critical limb ischaemia was considered. A range of studies have examined the question of quality of life because it is unclear which questions best describe quality of life.^11^-^15^ To evaluate quality of life, we used the SF-36, which has a generic and general meaning, and the VASCUQOL, which is specific to PAD.

The advantage of generic tests is that they can be used for a variety of diseases. However, their susceptibility is low, and their focus on specific effects related to the disease is not good.^16^ The VASCUQOL includes specific questions associated with PAD and better evaluates the effects after treatment.^17^,^18^ However, to date, no adequate comparative studies associated with generic or specific tests have been performed in patients with PAD. Comparative studies are needed. In the study performed by Morgan *et al.*, these two scores complemented each other and were compatible.^17^

Generally, it follows that when ischaemic lesions become serious in PAD, quality of life is impaired. In our study, impairment in the pain component of quality of life in Buerger’s disease patients was demonstrated. Buerger’s disease patients were more affected in terms of pain in both the SF-36 and VASCUQOL than were ASO patients. In an intergroup comparison, a statistically significant difference was found between the two groups in terms of pain at the time of hospital admission and at six months. Pain was identified to be more pronounced in Buerger’s disease patients; however, this significant difference disappeared at 12 months.

From an evaluation of all the heterogeneous patients included in this study, who had symptomatology varying from claudication to ischaemic gangrenes, Buerger’s disease generally impaired quality of life to a greater extent than ASO. Critical leg ischaemia was present in only half of the atherosclerotic patients whereas it was present in almost all of the Buerger’s disease patients.

In order to reduce heterogeneity in the evaluation, quality of life was evaluated in patients with only critical ischaemia. The conclusion was reached that the patient groups were not very different. In other words, when evaluating criteria such as pain at rest, or presence of ischaemic wound or gangrene, these conditions affected quality of life negatively, irrespective of whether they arose from ASO or Buerger’s disease.

Another parameter affecting quality of life was amputation. In many studies, amputations have been reported to seriously impair quality of life.^19^,^20^ In the study by Luther,20 144 patients with critical ischaemia were evaluated in terms of quality of life, determined by ankle/brachial index and pain. They also found that single or multiple amputations did not cause a difference in quality of life. Amputations were observed to affect morbidity and mortality rates however, particularly in the eighth and ninth decades.^20^

In our study, 23.4% of all patients with ASO and 48.7% of all patients with Buerger’s disease underwent amputation in the follow-up period of one year. Throughout the follow up, the quality of life in patients with amputations was significantly affected in terms of general health, pain, mental status, physical function, and emotional and social status in both ASO and Buerger’s disease patients.

According to the SF-36 score, amputation was observed to negatively affect pain at the time of admission in both ASO and Buerger’s disease patients. The quality of life in amputee patients was evaluated again at the six- and 12-month follow up. Amputations affected physical function, physical status, pain, general health, social function and emotional status in both ASO and Buerger’s disease patients.

When the difference in quality of life between amputee ASO and Buerger’s disease patients was evaluated with the VASCUQOL score, we observed no differences between the two groups, either at the time of hospital admission or at six or 12 months. The interpretation of this result is that because the source of the pain in patients who had undergone amputation due to either ASO or Buerger’s disease was removed, no difference in quality of life would be expected. However, quality of life was worse in Buerger’s disease patients without amputation compared with ASO patients.

There are a few limitations to this study. According to our study, Buerger’s disease affected quality of life more negatively than ASO, particularly with regard to pain score. Depressive symptoms are high among patients with PAD. The presence of depression may be a helpful factor in assessing pain scales in patients with PAD.^21^-^23^ However, in our study, patients’ psychological conditions were not considered. Assessment of life of quality may be more effective in PAD patients after treatment of psychological or emotional disorders.

Another limitation is that the effects of surgical and medical treatment on quality of life were not evaluated. Furthermore, as is commonly known, smoking is a highly significant risk factor for the progression of Buerger’s disease.^24^ However we did not compare quality of life between patients who had stopped smoking and those who continued to smoke. If the three parameters: determination of psychological situation, its treatment, and smoking had been taken into account, different results in terms of quality of life may have been observed.

## Conclusion

Buerger’s disease showed a more pronounced negative effect on quality of life than ASO, particularly in terms of pain score. However, when critical limb ischaemia was considered, both ASO and Buerger’s disease impaired the quality of life in patients to a similar degree. There is a need for further studies to reassess the quality of life after psychological assessment and treatment, where necessary, of patients.
