# Erratum

**Published:** 2023

The following article was published in the Cardiovascular Journal of Africa, volume 25, no 3, May/June 2014.

**Comparison of quality of life in patients with peripheral arterial disease caused by atherosclerosis obliterans or Buerger’s disease**

The authors were Rojbin Karakoyun, Cüneyt Köksoy, Zeynep Şener, Umut Gündüz, Bariş Karakaş, Mustafa Karakoyun.

The name of the mentor to Zeynep Şener, Semih Baskan, was omitted in error.

